# Finite element analysis of feeding in red and gray squirrels (*Sciurus vulgaris* and *Sciurus carolinensis*)

**DOI:** 10.1002/ar.25564

**Published:** 2024-08-21

**Authors:** Philip G. Cox, Peter J. Watson

**Affiliations:** ^1^ UCL Centre for Integrative Anatomy, Department of Cell and Developmental Biology University College London London UK; ^2^ Institute of Medical and Biological Engineering, School of Mechanical Engineering University of Leeds Leeds UK

**Keywords:** feeding biomechanics, finite element analysis, skull, squirrel

## Abstract

Invasive gray squirrels (*Sciurus carolinensis*) have replaced the native red squirrel (*Sciurus vulgaris*) across much of Great Britain over the last century. Several factors have been proposed to underlie this replacement, but here we investigated the potential for dietary competition in which gray squirrels have better feeding performance than reds and are thus able to extract nutrition from food more efficiently. In this scenario, we hypothesized that red squirrels would show higher stress, strain, and deformation across the skull than gray squirrels. To test our hypotheses, we created finite element models of the skull of a red and a gray squirrel and loaded them to simulate biting at the incisor, at two different gapes, and at the molar. The results showed similar distributions of strains and von Mises stresses in the two species, but higher stress and strain magnitudes in the red squirrel, especially during molar biting. Few differences were seen in stress and strain distributions or magnitudes between the two incisor gapes. A geometric morphometric analysis showed greater deformations in the red squirrel skull at all bites and gapes. These results are consistent with our hypothesis and indicate increased biomechanical performance of the skull in gray squirrels, allowing them to access and process food items more efficiently than red squirrels.

## 1 INTRODUCTION

The Eurasian red squirrel, *Sciurus vulgaris*, is distributed across the entire Palaearctic (Lurz et al., 2005), the largest range of all species of *Sciurus*. The subspecies *S. v. leucourus* is native to the British Isles, having arrived across the land bridge that existed between Great Britain and mainland Europe following the last glacial period (Barratt et al., 1999). Genetic studies have subsequently shown that the majority of extant populations of British *S. vulgaris* are of continental European subspecies ancestry (Hale et al., 2004). The British red squirrel population has fluctuated greatly over time: although probably widely distributed in the late medieval period, red squirrels suffered a steep decline in the 18th century, becoming almost completely extinct in Scotland, and locally extinct in parts of England and Wales (Lloyd, 1983). This dramatic decrease in numbers has been attributed to extensive woodland destruction, a colder climate, and possibly disease (Gurnell, 1987). The decline was arrested by numerous re‐introductions of red squirrels to Scotland from England and Scandinavia from the 1770s through the 1870s (Lowe & Gardiner, 1983). Red squirrels reached their peak distribution in Great Britain around the turn of the 20th century. From this high point, their numbers began to decrease and their range started to contract, slowly at first and then more steeply from the 1920s onwards (Gurnell, 1987; Shorten, 1954). This serious decline in red squirrels over the 20th century has been attributed to loss of woodland habitat and to competition with the invasive eastern gray squirrel (*S. carolinensis*). Gray squirrels were introduced to Britain from North America between 1876 and 1920 (Gurnell, 1987). Although initially slow to spread, gray squirrels expanded their range dramatically between 1930 and 1945 and are now found in almost every part of England and Wales, and much of Scotland. In contrast, red squirrels have disappeared from almost all of mainland England (except Northumberland and Cumbria) and Wales, as well as the central belt of Scotland (Crawley et al., 2020).

Various theories have been proposed to explain how gray squirrels are able to out‐compete reds so successfully (Gurnell & Pepper, 1993). Disease appears to be a major factor: gray squirrels are known to be carriers of squirrelpox virus, which does not seem to affect them adversely, but which can be fatal to red squirrels (LaRose et al., 2010; Tompkins et al., 2002, 2003). In addition, a spillover of helminth parasites from gray to red squirrels has also been noted (Romeo et al., 2015). However, it has been shown that the gray squirrel still causes the decline of the red squirrel in the absence of disease (Gurnell et al., 2004; Rushton et al., 2006; Wauters et al., 2000, 2001, 2002, 2023). Low fecundity in red squirrels compared to gray squirrels (MacKinnon, 1978), and decreased growth rates and survival rates of juvenile red squirrels when in the presence of gray squirrels (Gurnell et al., 2004; Harris & Yalden, 2008; Wauters et al., 2000, 2023), also appears play a part. Although direct aggression by gray squirrels toward red squirrels has been suggested (Bertram & Moltu, 1986; MacKinnon, 1978), it does not seem to be a factor (Gurnell & Pepper, 1993), and indeed direct interactions between red and gray squirrels are rarely observed (Wauters et al., 2023) despite considerable overlap of their ranges in northern England and the southern half of Scotland (Dunn et al., 2021). Notably, the decline of red squirrels has not been regionally uniform across Britain. Red squirrels have been able to persist longer in woodland with a high proportion of conifers (e.g., Cannock Chase, Thetford Chase, Clocaenog Forest, Kielder Forest; Crawley et al., 2020), indicating that there are environments in which red squirrels are better able to compete against grays. Direct competition for food is likely to play a role, with gray squirrels able to exploit resources more efficiently than red squirrels (Gurnell & Pepper, 1993) and gray squirrels being better equipped physiologically for the digestion of toxic acorns (Kenward & Holm, 1993). Such dietary competition is known to be a major factor influencing species distribution and survival in many vertebrates (Brown et al., 2014; Hayward & Kerley, 2008; Kamilar & Ledogar, 2011; Mitchell & Banks, 2005), but whether red and gray squirrels differ in their abilities to mechanically access and break down dietary items is as yet unknown.

In this study, the aim is to investigate the potential for differences in biting ability between red and gray squirrels using finite element analysis (FEA), a virtual simulation technique originally developed in the field of engineering, but now frequently used in functional morphology to address biomechanical hypotheses (Rayfield, 2007). FEA simulates the response of an object with complex geometry to a static load, and is able to predict the stresses, strains, and deformations experienced in that scenario. It has been employed extensively in vertebrate biology to model the impact of the forces generated by the masticatory musculature during feeding on the skull (e.g., Figueirido et al., 2014; Godinho et al., 2017; McIntosh & Cox, 2016; Penrose et al., 2020; Ruiz et al., 2023; Sharp et al., 2023; Tseng, 2009; Watson et al., 2021). Here, FEA will be used to compare the biomechanical response of red and gray squirrel skulls during incisor gnawing and molar chewing. We hypothesize that gray squirrels will show better feeding performance than red squirrels based on the grays' ability to outcompete and replace reds in Britain. As in other FE studies, “better performance” will be defined as displaying (a) lower strain values—these indicate a skull that is better adapted to the forces being generated by the muscles, under the bone functional adaptation hypothesis (Ruff et al., 2006)—and (b) lower von Mises stresses—this will increase the safety factor of the skull by decreasing the likelihood that stresses will approach the yield strength of bone.

## 2 MATERIALS AND METHODS

### 2.1 Sample and model creation

Three‐dimensional reconstructions of the skulls of an adult red and gray squirrel previously created for multibody dynamics analysis (Cox & Watson, 2023) were reused in this study. The red squirrel skull was 42.8 mm in length, which is within the range reported by Lurz et al. (2005) for this species. The gray squirrel skull was 48.2 mm which is relatively small: Koprowski (1994) reports a mean greatest skull length of 60.7 mm (but no range or standard deviation is given). Thus, the size difference between these models slightly under‐represents the typical species‐level size difference (as reported by e.g., Bryce et al., 2001). Cranial reconstructions were created in AVIZO v.2020.3 (Thermo Fisher Scientific, Waltham, MA, USA) from microCT scans with isometric voxel dimensions of 0.030 mm (red) and 0.066 mm (gray). The two microCT scans are freely available for download from www.morphosource.org (gray squirrel doi: 10.17602/M2/M47051; red squirrel doi: 10.17602/M2/M488419). Bone and teeth were segmented as separate materials. Volumetric models of each skull were meshed in AVIZO using second order tetrahedral elements, creating meshes with a total of ~4.35 million elements and ~4.75 million elements for the red and gray squirrel, respectively.

### 2.2 Model inputs

The meshes of both skulls were imported to ANSYS v.23 (ANSYS Inc., USA) and all materials were defined with homogenous, isotropic, and linear elastic properties. Based on previous nano‐indentation work (Cox et al., 2011, 2012), bone and teeth were assigned Young's moduli of 17 and 30 GPa respectively. Both materials were assigned a Poisson's ratio (*ν*) of 0.3. Following previous FE analyses (Cox et al., 2012; Watson et al., 2021), both models were constrained in the following manner when simulating a maximal bilateral incisor bite: a node at the left temporo‐mandibular joint (on the ventral surface of the zygomatic process of the squamosal where the mandibular condyle would articulate) was constrained in all degrees of freedom; a node at the right temporo‐mandibular joint was constrained in the dorso‐ventral and antero‐posterior directions; and a node on the occlusal surface of each incisor was constrained in the dorsoventral direction. For comparative purposes, simulation of a maximal molar bite used the same temporo‐mandibular joint constraint configurations and constrained a node on the occlusal surface of the right third molar in the dorsoventral direction.

Forces were applied to each model to simulate the action of the following muscles: superficial masseter, anterior and posterior deep masseter, anterior and posterior zygomaticomandibularis, temporalis, medial, and lateral pterygoid. The location and magnitude of muscles forces applied to both models when simulating maximum bilateral incisor biting were in accordance with a previous multibody dynamics analysis of the red and gray squirrel (Table 1; see also fig. 1 in Cox & Watson, 2023). The muscle attachment areas were determined via dissection (Cox & Watson, 2023) and previously published descriptions (Ball & Roth, 1995; Cox & Jeffery, 2011; Druzinsky, 2010). A further multibody dynamics analysis was performed to predict the magnitude of the muscle forces associated with a maximum bite on the right third molar (Table 1). Following the multibody dynamics analysis of incisor biting (Cox & Watson, 2023), simulation of the molar biting modeled the temporo‐mandibular as a revolute joint (only permitting rotation in one degree of freedom).

**TABLE 1:** Muscle forces (N) applied to each side of each finite element model.

Muscle	Gray squirrel	Red squirrel
Superficial masseter	10.3	6.7
Anterior deep masseter	8.8	7.1
Posterior deep masseter	9.5	6.8
Anterior zygomaticomandibularis	6.9	2.5
Posterior zygomaticomandibularis	2.0	3.0
Temporalis	4.2	5.7
Medial pterygoid	10.3	5.0
Lateral pterygoid	3.3	3.7

### 2.3 Model solution and analysis

The FEA models were solved for three biting scenarios—two maximum bilateral bites at the incisors at different gapes (7.5 and 15 mm), and a unilateral maximum bite on the right third molar (at 2 mm gape). Gapes were defined as linear distances rather than angles so that the mechanical response to biting on food items of equivalent size could be compared. The largest gape (15 mm) was chosen to represent the diameter of an average‐sized hazelnut. The bite force generated in each scenario was calculated by the multibody dynamics analysis simulations (Cox & Watson, 2023). Mechanical advantage was calculated by dividing the bite force by the total input adductor muscle force. Principal strains (*ε*) 1 and 3 (representing predominantly tensile and predominantly compressive strains respectively) and von Mises stresses were visualized across the loaded skull models as contour maps. In order to compare the overall strain experienced by each skull in a quantitative manner, the cranial deformations experienced during biting were analyzed using geometric morphometric methods (O'Higgins et al., 2011, 2012). For this a set of 44 3D landmark co‐ordinates (Table S1 and Figure S1) were recorded from the unloaded skull and from each of the three loading scenarios for both the red and gray squirrel models. As the differences between the two unloaded models would far outweigh the differences between loaded and unloaded models, the procedure set out in McIntosh and Cox (2016) was followed to facilitate comparisons between the red and gray squirrel crania. Landmarks were subjected to a generalized Procrustes analysis and scaled to centroid size. The mean landmark configuration of the unloaded red and gray squirrel models was calculated, and the residual landmark displacements between the loaded and unloaded models were added to this mean configuration. A second Procrustes analysis was then performed on the mean unloaded landmark set and the new loaded landmark configurations, this time without scaling or tangent projection (O'Higgins & Milne, 2013). Principal components analysis was used to visualize the results in a morphospace in which the vector of each loaded model from the mean unloaded model represented the mode and magnitude of deformation experienced during each biting scenario. Geometric morphometric analyses were conducted using MorphoJ v.1.07a (Klingenberg, 2011) and the EVAN toolbox v.1.75 (www.evan-society.org).

## 3 RESULTS

**FIGURE 1:**
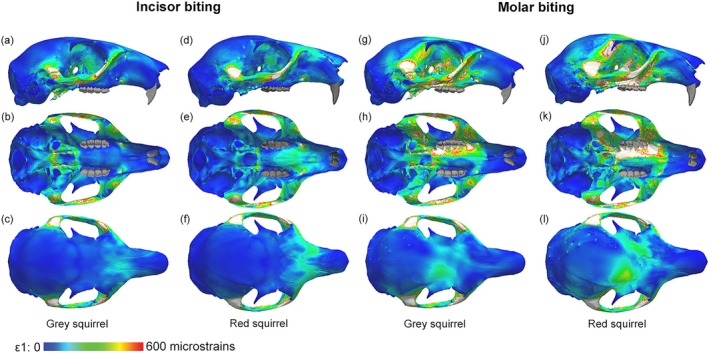
Contour maps showing the distribution of maximum principal strains across the skull predicted by FEA during biting at the (a–f) incisor and (g–l) molar in (a–c, g–i) gray and (d–f, j–l) red squirrels. Views: (a, d, g, j) right lateral, (b, e, h, k) ventral, and (c, f, i, l) dorsal. Warm colors represent high strains, cool colors represent low strains. White areas represent elements experiencing greater than 600 microstrains.

**FIGURE 2:**
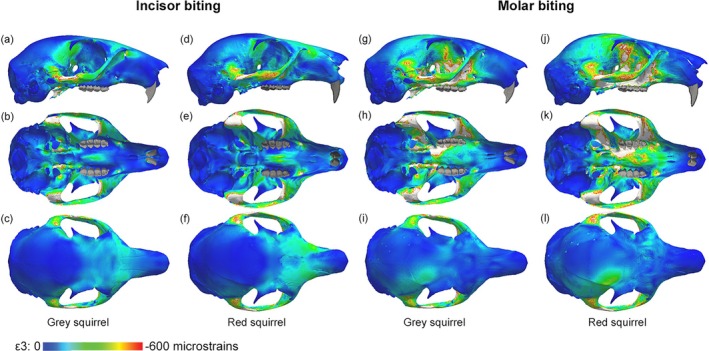
Contour maps showing the distribution of minimum principal strains across the skull predicted by FEA during biting at the (a–f) incisor and (g–l) molar in (a–c, g–i) gray and (d–f, j–l) red squirrels. Views: (a, d, g, j) right lateral, (b, e, h, k) ventral, and (c, f, i, l) dorsal. Warm colors represent low strains, cool colors represent high strains. White areas represent elements experiencing less than −600 microstrains.

**FIGURE 3:**
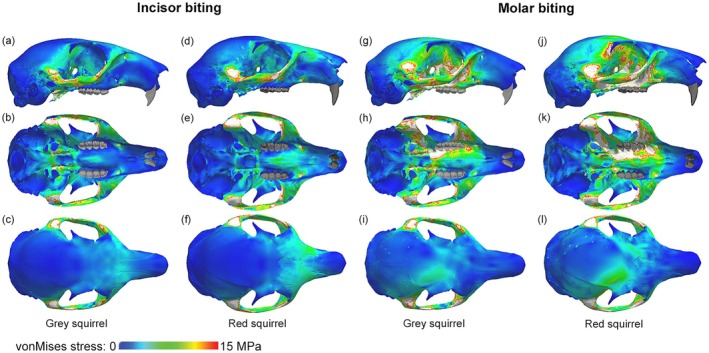
Contour maps showing the distribution of von Mises stresses across the skull predicted by FEA during biting at the (a–f) incisor and (g–l) molar in (a–c, g–i) gray and (d–f, j–l) red squirrels. Views: (a, d, g, j) right lateral, (b, e, h, k) ventral, and (c, f, i, l) dorsal. Warm colors represent high stresses, cool colors represent low stresses. White areas represent elements experiencing greater than 15 MPa.

The predicted distribution of principal strains across red and gray squirrel skulls during maximum incisor biting at a 7.5 mm gape between the incisor tips can be seen in Figures 1 and 2. The distribution patterns were similar, though not identical, between the two species, with high strains seen along the zygomatic arch from the temporo‐mandibular joint to the zygomatic plate, and in the orbit and orbito‐temporal region. In the red squirrel skull, *ε*1 was increased across the palate between the tubercles where the superficial masseter muscles attach and as far back as the region between the first molars (Figure 1). In the gray squirrel skull, *ε*1 values were lower in this region, but higher more posteriorly along the cranial base, around the pterygoid processes. Similar differences existed between the *ε*3 distributions: the red squirrel skull showed higher *ε*3 values across the dorsal surface of the skull between the zygomatic plates, whereas the gray squirrel model had higher *ε*3 values in the posterior part of the orbit and anterior temporal region (Figure 2). The von Mises stress distributions were very similar to the principal strain distributions in both the red and gray squirrel skulls. Overall, the red squirrel skull showed slightly higher von Mises stresses, with the regions showing the highest stresses being enlarged compared to the gray squirrel skull (Figure 3). The predicted bite force in the gray squirrel was around 1.3 times greater than in the red squirrel (Table 2), but the mechanical advantage was almost identical in the two models (~0.30).

**TABLE 2:** Bite force and mechanical advantage (bite force divided by total adductor muscle force) predicted by each finite element model.

Bite point	Gape (mm)	Bite force (N)		Mechanical advantage	
		Gray squirrel	Red squirrel	Gray squirrel	Red squirrel
Incisor	7.5	33.29	24.67	0.301	0.305
Incisor	15	38.77	26.95	0.351	0.333
Molar	2	75.11	56.06	0.679	0.692

The red and gray squirrel skull models were also solved for incisor biting at a wider gape of 15 mm between the incisor tips. Despite differences in the line of action of each muscle and bite force magnitude, very few differences in strain and stress distributions across the skull could be identified between the two incisor gape scenarios. Contour maps showing the distributions of maximum principal strains, minimum principal strains, and von Mises stress are provided in Figures S2–S4 for both gapes and both models. Bite forces at the wider 15 mm gape were larger than at the narrower gape by 2.28 N in the red squirrel and 5.48 N in the gray squirrel (Table 2). Mechanical advantage was also larger at the wider gape in both squirrels, although once again there was little difference seen between the two species (0.35 in the gray squirrel vs. 0.33 in the red squirrel).

Maximum molar biting generated notably higher strains and stresses when compared to maximum incisor biting. As above, the distributions across the skull were similar between the red and gray squirrel skulls, but the strain and stress magnitudes were higher in the red squirrel model. High *ε*1 values were concentrated around the working side orbit, orbito‐temporal region, and palate, as well as both zygomatic arches (Figure 1). There was also an area of elevated strain on the dorsal cranial surface in the interorbital region. This was restricted to the working side in the gray squirrel model but extended to the balancing side in the red squirrel model, forming a helical pattern across the skull. The *ε*3 values have a similar pattern, but with higher strains across the temporal fossa and over the posterior‐most part of the palate (Figure 2). Von Mises stress distributions (Figure 3) told a similar story to the principal strains and clearly showed increased stress in the red squirrel skull compared to the gray squirrel. As expected, the predicted bite force at the molars was just over double that at the incisors, with the gray squirrel generating around 30% more bite force at the molars than the red squirrel (56.06 N in the red squirrel and 75.11 N in the gray squirrel), as also seen at the incisors (Table 2). Mechanical advantage at the molars was very similar between the two species (0.68 in the gray squirrel and 0.69 in the red squirrel).

**FIGURE 4:**
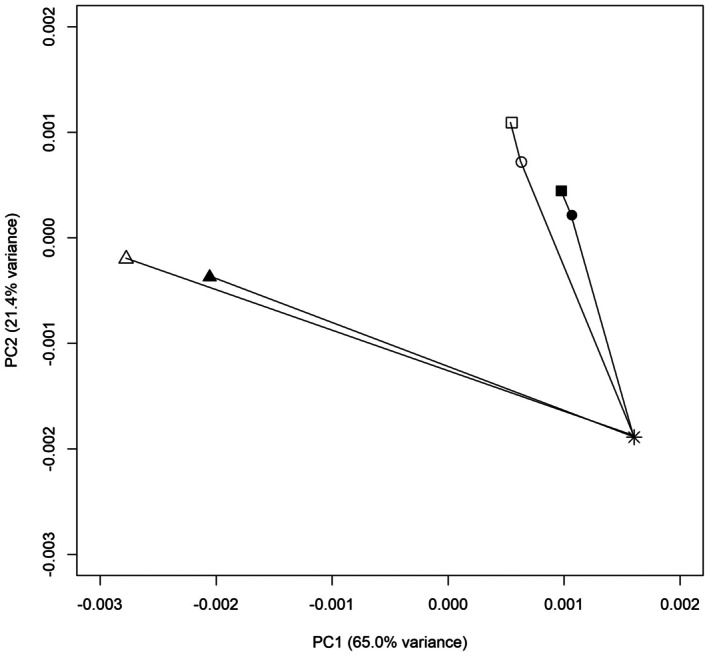
Plot of first two principal components representing the differences in cranial deformations between the three loading scenarios in each model. Lines represent trajectories of deformation. Star, mean unloaded model; open shapes, red squirrel models; filled shapes, gray squirrel models; squares, incisor biting at 7.5 mm gape; circles, incisor biting at 15 mm gape; triangles, molar biting.

The six loading scenarios were compared to the mean unloaded model using geometric morphometrics. The first two principal components (representing over 88% of the total variance) are shown in Figure 4, and Procrustes distances between unloaded model and each load case are given in Table 3. Although the strain distributions were similar for the red and gray squirrel models during incisor biting, the morphometrics confirmed that the red squirrel skull experienced greater deformation than the gray squirrel skull in all three biting scenarios. The way in which the skulls deformed was clearly very different between bites as shown by the wide angle between the trajectories for incisor biting and molar biting. The deformation trajectories for molar biting were very similar in the two squirrel species, whereas a larger angle was seen between the deformation trajectories of incisor biting indicating more distinct modes of deformation between red and gray squirrels. Despite the lack of visible differences in strain patterns between the two incisor gapes within each model, Figure 4 shows that biting at the narrower gape (7.5 mm) generated greater cranial deformation than the wider gape (15 mm).

**TABLE 3:** Procrustes distances between the mean unloaded model and each loaded model.

Bite point	Gape (mm)	Procrustes distance	
		Gray squirrel	Red squirrel
Incisor	7.5	0.000267	0.000317
Incisor	15	0.000237	0.000279
Molar	2	0.000422	0.000472

## 4 DISCUSSION

The results of the FEA modeling in this study predicted similar patterns of principal strains and von Mises stresses across the skull of red and gray squirrels during biting at the incisors and at the cheek teeth. However, the red squirrel skull generally experiences higher strains and stresses, and greater deformation, than the gray squirrel skull, consistent with our original hypothesis that the red squirrel skull performs less well than the gray squirrel skull when feeding. Predicted bite forces are higher in the gray squirrel skull, consistent with its larger size and larger masticatory muscles (Cox & Watson, 2023). However, only negligible differences were found in mechanical advantage between the two species, indicating that the gray squirrel masticatory apparatus is not more efficient at producing bite force than that of the red squirrel.

During incisor biting, the red squirrel skull shows high *ε*1 values across the palate anterior to the cheek teeth (Figure 1), and high *ε*3 values across the dorsal surface of the skull anterior to the orbits (Figure 2). As maximum principal strains are predominantly tensile and minimum principal strains are predominantly compressive, this suggests that biting at the incisors is deforming the rostrum superiorly relative to the braincase just in front of the orbit. This effect is much less pronounced in the gray squirrel model, probably because its skull is slightly taller in the antorbital region, which better resists the ventral pull of the jaw‐closing muscles. A similar effect has been noted in the domed skulls of durophagous carnivorans which exhibit lower stresses in the frontal region compared to relatives with flatter skulls (Figueirido et al., 2014; Tseng, 2009; Tseng & Wang, 2010).

The other major difference between the models during incisor biting is that the gray squirrel seems to have slightly higher strains and von Mises stresses in the posterior regions of the skull. This is most notable in the orbito‐temporal region (Figure 2) and cranial base (Figures 1 and 3). This suggests that the resultant vector representing the overall magnitude and direction of contraction of the masticatory muscles when simultaneously activated (Greaves, 1978) is situated more posteriorly in the gray squirrel compared to the red. In particular, the elevated cranial base strains may be a result of the proportionally larger medial pterygoid in the gray squirrel (Cox & Watson, 2023). However, despite the elevated strains in the posterior part of the gray squirrel skull, when taken as a whole, the red squirrel skull experiences greater deformation during incisor biting (Figure 4), indicating poorer performance during this activity.

It can be seen from the morphospace in Figure 4 that incisor biting at a narrower gape generates higher levels of deformation than at a wider gape in both species. This result is consistent with a skull morphology that has adapted, via either selection or bone modeling/remodeling, to feeding at wide gapes, as would be needed to deal with the relatively large and mechanically resistant food items (e.g., hazelnuts and acorns) often found in squirrel diets (Moller, 1983; Wauters et al., 2001).

Molar bites result in increased maximum strains and stresses across the skull, as noted in other previous studies of rodents (Cox et al., 2011, 2012). This is also expected from lever‐arm mechanics as the position of the molar bite point, closer to the jaw joint tends to concentrate the muscle load over a smaller proportion of the skull, leaving the rostrum largely unloaded, compared to an incisor bite. Again, the gray squirrel model performs better (i.e., experiences lower strains and stresses) compared to the red squirrel (Figure 4). The asymmetrical pattern of strains across the cranial vault (Figure 1) suggests that the skull is experiencing torsional forces during unilateral molar biting and that these are more pronounced in the red squirrel skull. Such helical patterns of strain were predicted in response to unilateral bites by Greaves (1991) and Covey and Greaves (1994) and represent the rotation of the anterior and posterior portions of the skull in opposite directions owing to reaction forces at the biting tooth and balancing side jaw joint. Elevated strains along these torsional arcs have been recovered in other FEA studies (Mitchell, 2019).

The increased principal strains across the red squirrel skull during both incisor and molar biting, alongside the geometric morphometric analysis, indicate that the gray squirrel skull deforms less during maximal biting. It should also be noted that the maximum bite forces modeled in these analyses are higher in the gray squirrel than the red squirrel (around 1.3 times greater, Table 1) owing to the larger size of the gray squirrel skull and masticatory muscles. If the red squirrel bite force were scaled up to that of the gray squirrel, the principal strain values in the red squirrel skull would be approximately 1.3 times greater because strain magnitudes scale linearly with force if material properties are linearly elastic and loads are static (Toro‐Ibacache et al., 2016), further increasing the difference between the red and gray squirrel models. Therefore, these results suggest that gray squirrels are better adapted than red squirrels to generating bite forces closer to their maximum. Such adaptation could result from natural selection for higher bite forces over many generations, or from bone remodeling within the lifetime of individual squirrels in response to dietary mechanical properties (bone functional adaptation: Cowin et al., 1985; Lanyon & Rubin, 1985; Ruff et al., 2006). Rodent masticatory morphology has been shown to undergo rapid evolutionary change in response to a novel food source (Doudna & Danielson, 2015), and also to remodel in response to altered dietary consistency in laboratory experiments (e.g., Anderson et al., 2014; Mitchell et al., 2021; Ödman et al., 2008). Thus both mechanisms are likely to have played a role in driving the morpho‐functional changes identified in this study.

Alongside decreased strains, it is also shown here that the gray squirrel skull experiences lower von Mises stress than the red squirrel skull, which increases the safety factor during biting and reduces the risk of bone failure, allowing higher bite forces to be generated safely. Furthermore, gray squirrels are generally larger than reds (Bryce et al., 2001) and can produce higher bite forces. Thus, gray squirrels are able to eat harder or tougher examples of dietary items than red squirrels. This will enable them to access a wider proportion of each nut species available in their environment, which will be particularly advantageous in mixed broadleaf woodland over winter when the diet of both squirrels is largely high‐energy, but difficult to access, hazelnuts, beechnuts, chestnuts, and acorns (Wauters et al., 2023). This wider feeding envelope may be a contributing factor to gray squirrels having been able to outcompete red squirrels so widely across Great Britain, as suggested previously (Gurnell & Pepper, 1993).

The difference in strain and stress values between the red and gray squirrel models is even greater in the molar biting scenario than incisor biting (Figures 1, 2, 3). This suggests that any competitive advantage conferred by increased feeding performance would have greater impact when eating food items that require processing at the cheek teeth. Thus, there may only be a small difference in the relative ability of red and gray squirrels to feed on pine seeds because they presumably require very little molar processing, being so small. The bulk of the effort in eating pine seeds is expended accessing the seeds by removing the scales from the cones—an activity undertaken exclusively via incisor gnawing. In contrast, feeding on items such as hazelnuts and acorns requires both incisor gnawing to remove the shell and then molar chewing to break down the large edible kernel. The enhanced ability of gray squirrels to process food at the molars is consistent with their known preference for deciduous seeds compared to red squirrels which prefer conifer seeds (Moller, 1983). It may also have played a role in red squirrels persisting for longer in areas with a high proportion of coniferous trees such as Thetford Forest, Cannock Chase, and Clocaenog Forest compared to mixed and deciduous woodland (Crawley et al., 2020; Gurnell & Pepper, 1993).

## 5 CONCLUSIONS

The results of this study have revealed subtle, but visible, differences in the response of red and gray squirrel skulls to masticatory loads. Red squirrels experience higher strains and stresses during maximum bite force generation compared to gray squirrels, especially during molar biting. While there are clearly multiple factors underlying the replacement of red squirrels by gray squirrels in Britain, the results here hint that dietary competition and differences in feeding performance between the two species may play a role in the replacement and also in determining the areas in which red squirrels are able to persist.

## AUTHOR CONTRIBUTIONS

**Philip G. Cox:** Conceptualization; formal analysis; funding acquisition; investigation; methodology; writing – original draft; writing – review and editing. **Peter J. Watson:** Conceptualization; formal analysis; funding acquisition; investigation; methodology; writing – review and editing.

## FUNDING INFORMATION

This research was funded by APEX award APX\R1\201038 from the Leverhulme Trust, the British Academy, the Royal Society of Engineering and the Royal Society. MicroCT scanning of the gray squirrel skull was funded by NERC standard grant no. NE/G001952/1.

## Supporting information


**TABLE S1:** Cranial landmarks used in the GMM analyses. Landmarks are visualized in Figure S1. Landmarks 1–10 are in the midsagittal plane. Landmarks 11–27 were collected from the left side of the skull and landmarks 28–44 from the right side of the skull.
**FIGURE S1.** 3D landmark configuration recorded from red squirrel cranium shown on red squirrel specimen in (a) left lateral, (b) dorsal, and (c) ventral view. Landmarks 1–10 are in the midsagittal plane.
**FIGURE S2.** Contour maps showing the distribution of maximum principal strains across the skull predicted by FEA during incisor biting in (a–f) gray and (g–l) red squirrels at a gape of (a–c, g–i) 7.5 mm and (d–f, j–l) 15 mm. Views: (a, d, g, j) right lateral, (b, e, h, k) ventral, and (c, f, i, l) dorsal. Warm colors represent high strains, cool colors represent low strains. White areas represent elements experiencing greater than 600 microstrains.
**FIGURE S3.** Contour maps showing the distribution of minimum principal strains across the skull predicted by FEA during incisor biting in (a–f) gray and (g–l) red squirrels at a gape of (a–c, g–i) 7.5 mm and (d–f, j–l) 15 mm. Views: (a, d, g, j) right lateral, (b, e, h, k) ventral, and (c, f, i, l) dorsal. Warm colors represent low strains, cool colors represent high strains. White areas represent elements experiencing less than –600 microstrains.
**FIGURE S4.** Contour maps showing the distribution of von Mises stresses across the skull predicted by FEA during incisor biting in (a–f) gray and (g–l) red squirrels at a gape of (a‐c, g–i) 7.5 mm and (d–f, j–l) 15 mm. Views: (a, d, g, j) right lateral, (b, e, h, k) ventral, and (c, f, i, l) dorsal. Warm colors represent high stresses, cool colors represent low stresses. White areas represent elements experiencing greater than 15 MPa.
