# Supplementary material for: Finite element analysis of feeding in red and gray squirrels (Sciurus vulgaris and Sciurus carolinensis)
Source: Anat Rec (Hoboken). 2024 Aug 21;309(9):2476–86. doi: 10.1002/ar.25564 (PMC13431925; doi:10.1002/ar.25564)
Supplement: Supplementary file 1 — TABLE S1: Cranial landmarks used in the GMM analyses. Landmarks are visualized in Figure S1. Landmarks 1–10 are in the midsagittal plane. Landmarks 11–27 were collected from the left side of the skull and landmarks 28–44 from the right side of the skull. FIGURE S1. 3D landmark configuration recorded from red squirrel cranium shown on red squirrel specimen in (a) left lateral, (b) dorsal, and (c) ventral view. Landmarks 1–10 are in the midsagittal plane. FIGURE S2. Contour maps showing the distribution of maximum principal strains across the skull predicted by FEA during incisor biting in (a–f) gray and (g–l) red squirrels at a gape of (a–c, g–i) 7.5 mm and (d–f, j–l) 15 mm. Views: (a, d, g, j) right lateral, (b, e, h, k) ventral, and (c, f, i, l) dorsal. Warm colors represent high strains, cool colors represent low strains. White areas represent elements experiencing greater than 600 microstrains. FIGURE S3. Contour maps showing the distribution of minimum principal strains across the skull predicted by FEA during incisor biting in (a–f) gray and (g–l) red squirrels at a gape of (a–c, g–i) 7.5 mm and (d–f, j–l) 15 mm. Views: (a, d, g, j) right lateral, (b, e, h, k) ventral, and (c, f, i, l) dorsal. Warm colors represent low strains, cool colors represent high strains. White areas represent elements experiencing less than –600 microstrains. FIGURE S4. Contour maps showing the distribution of von Mises stresses across the skull predicted by FEA during incisor biting in (a–f) gray and (g–l) red squirrels at a gape of (a‐c, g–i) 7.5 mm and (d–f, j–l) 15 mm. Views: (a, d, g, j) right lateral, (b, e, h, k) ventral, and (c, f, i, l) dorsal. Warm colors represent high stresses, cool colors represent low stresses. White areas represent elements experiencing greater than 15 MPa. [file AR-309-2476-s001.pdf]

Table S1. Cranial landmarks used in the GMM analyses. Landmarks are visualised in Figure S1. Landmarks 1-10 are in the midsagittal plane. Landmarks 11-27 were collected from the left side of the skull and landmarks 28-44 from the right side of the skull.

**Cranial Landmarks**	
**1**	Anteriormost point on internasal suture
**2**	Posteriormost point on internasal suture
**3**	Midpoint of line running between dorsal orbital notches
**4**	Midpoint of coronal (fronto-parietal) suture
**5**	Posteriormost point on dorsal midline
**6**	Anterior midpoint of incisor alveoli
**7**	Posterior midpoint of incisor alveoli
**8**	Midpoint between anteriormost points of first premolar alveoli
**9**	Posteriormost midline point on palate
**10**	Midline point of the ventral margin of the foramen magnum
**11 & 28**	Anteriormost point on naso-premaxillary suture
**12 & 29**	Superiormost point on margin of infraorbital foramen
**13 & 30**	Anteriormost extremity of zygomatic plate
**14 & 31**	Postero-lateral extremity of zygomatic plate
**15 & 32**	Ventralmost point of lacrimal tubercule on orbital margin
**16 & 33**	Anteriormost point on alveolar margin of first premolar
**17 & 34**	Posteriormost point on alveolar margin of third molar
**18 & 35**	Medialmost point in superior orbital notch
**19 & 36**	Anteriormost point in notch between postorbital process and cranial vault
**20 & 37**	Extremity of postorbital process
**21 & 38**	Anteriormost point on margin of optic foramen
**22 & 39**	Superiormost point on dorsal prominence of zygomatic arch
**23 & 40**	Anterior extremity of zygomatic process of squamosal
**24 & 41**	Posteriormost point on anterior margin of zygomatic process of squamosal
**25 & 42**	Posteriormost point on jugal bone
**26 & 43**	Anteriormost point of auditory bulla
**27 & 44**	Ventral apex of auditory bulla

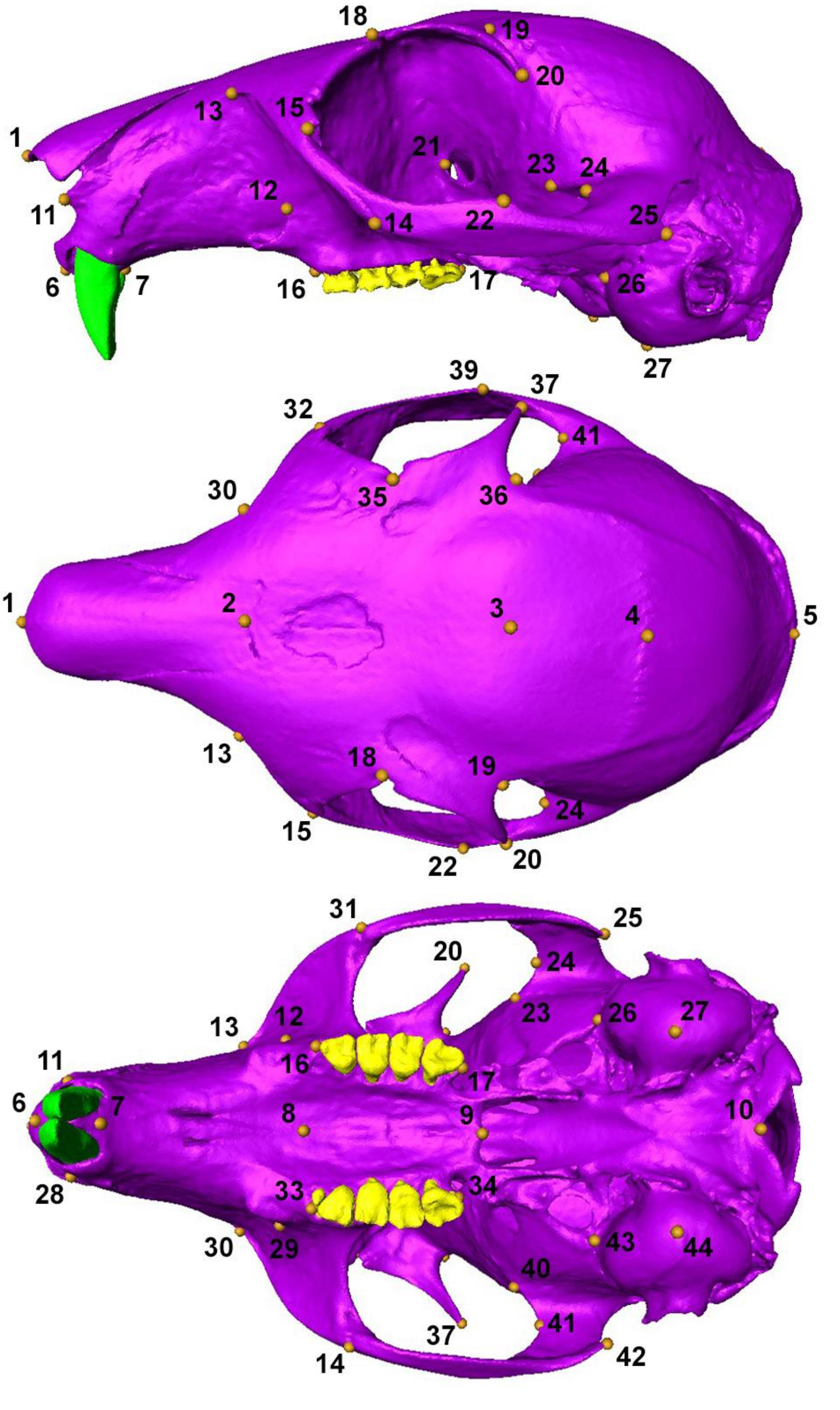

Figure S1. 3D landmark configuration recorded from red squirrel cranium shown on red squirrel specimen in (a) left lateral, (b) dorsal and (c) ventral view. Landmarks 1-10 are in the midsagittal plane.

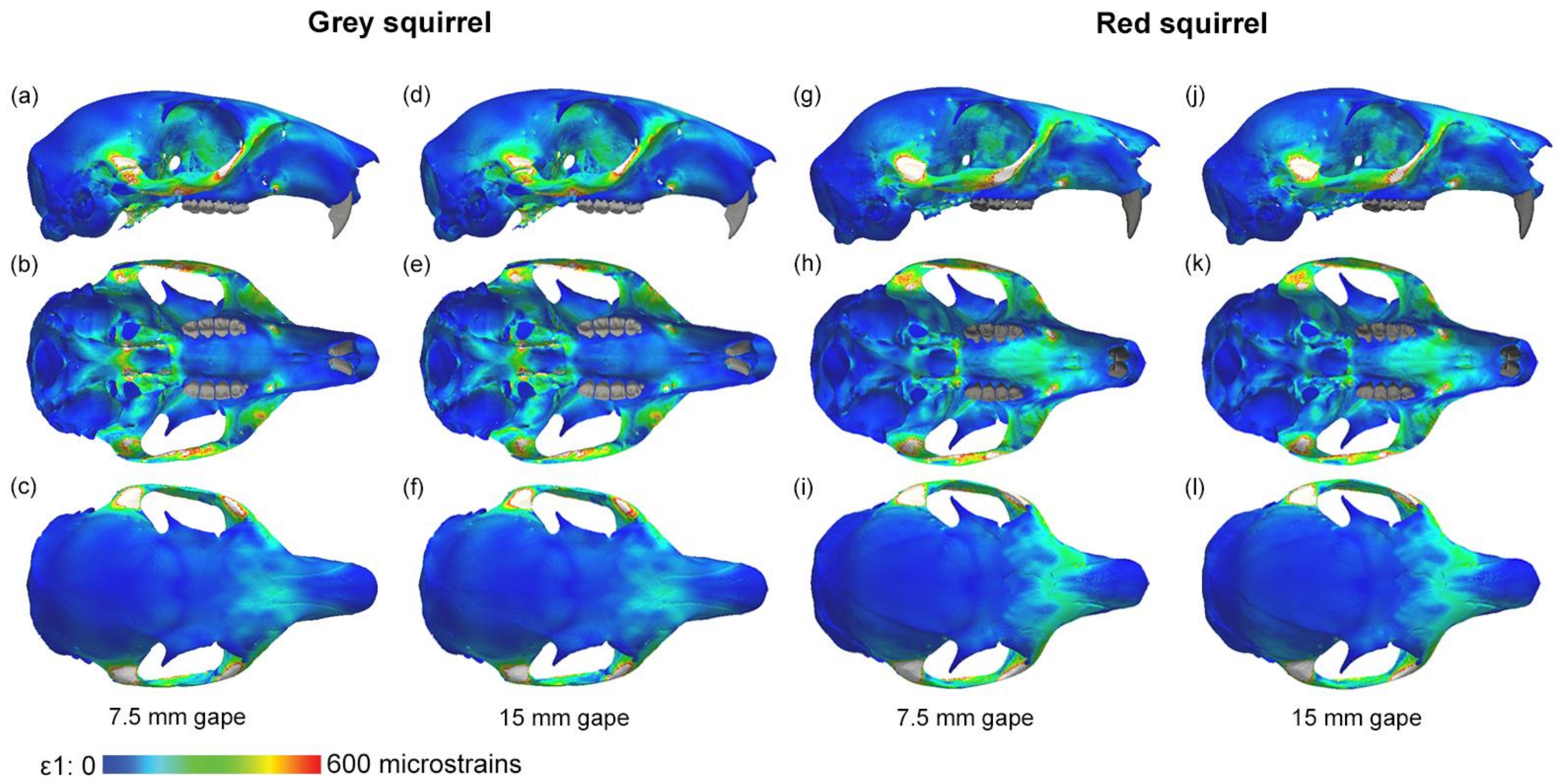

Figure S2. Contour maps showing the distribution of maximum principal strains across the skull predicted by FEA during incisor biting in (a-f) grey and (g-l) red squirrels at a gape of (a-c,g-i) 7.5 mm and (d-f,j-l) 15 mm. Views: (a,d,g,j) right lateral, (b,e,h,k) ventral, and (c,f,i,l) dorsal. Warm colours represent high strains, cool colours represent low strains. White areas represent elements experiencing greater than 600 microstrains.

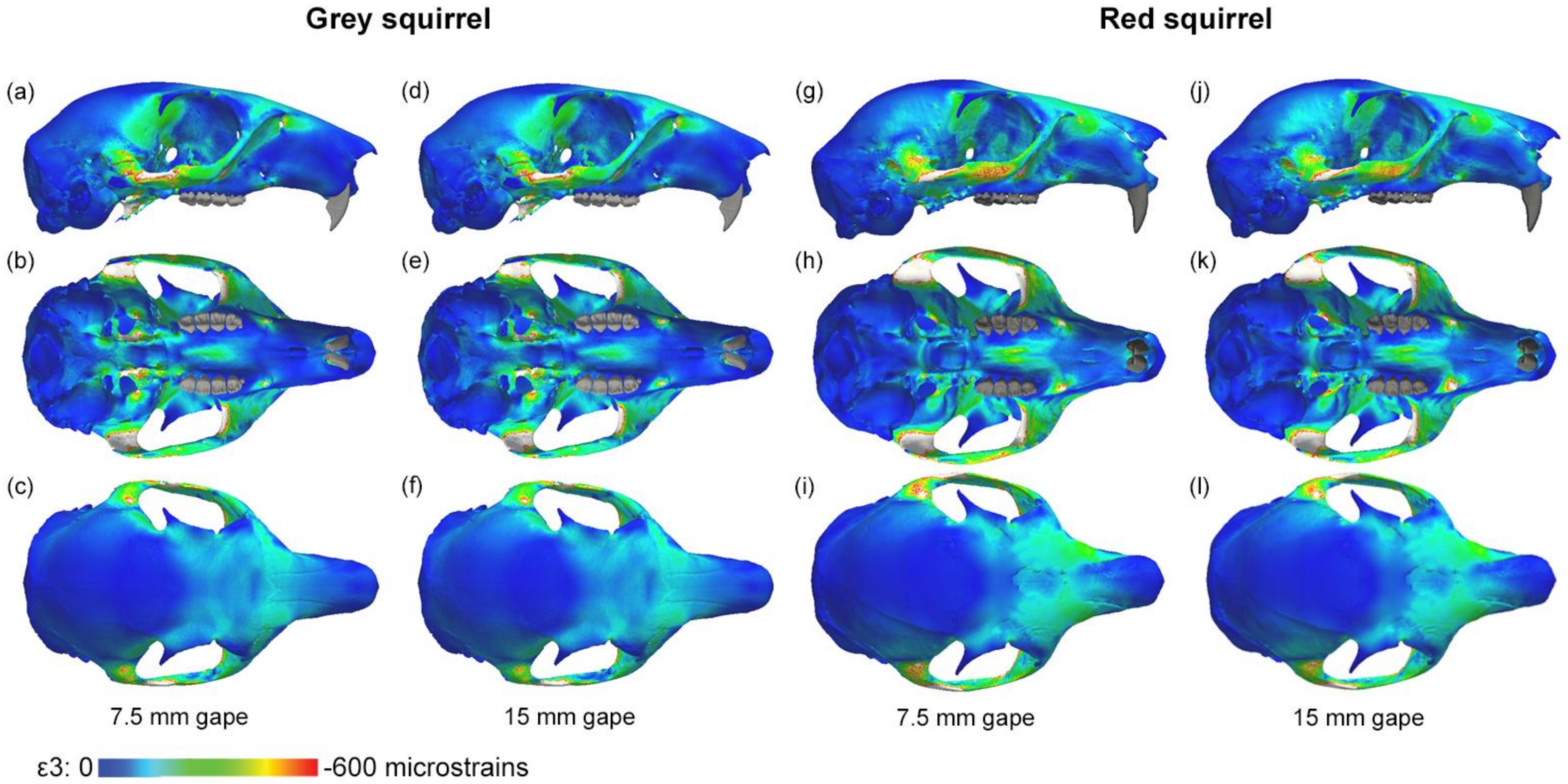

Figure S3. Contour maps showing the distribution of minimum principal strains across the skull predicted by FEA during incisor biting in (a-f) grey and (g-l) red squirrels at a gape of (a-c,g-i) 7.5 mm and (d-f,j-l) 15 mm. Views: (a,d,g,j) right lateral, (b,e,h,k) ventral, and (c,f,i,l) dorsal. Warm colours represent low strains, cool colours represent high strains. White areas represent elements experiencing less than -600 microstrains.

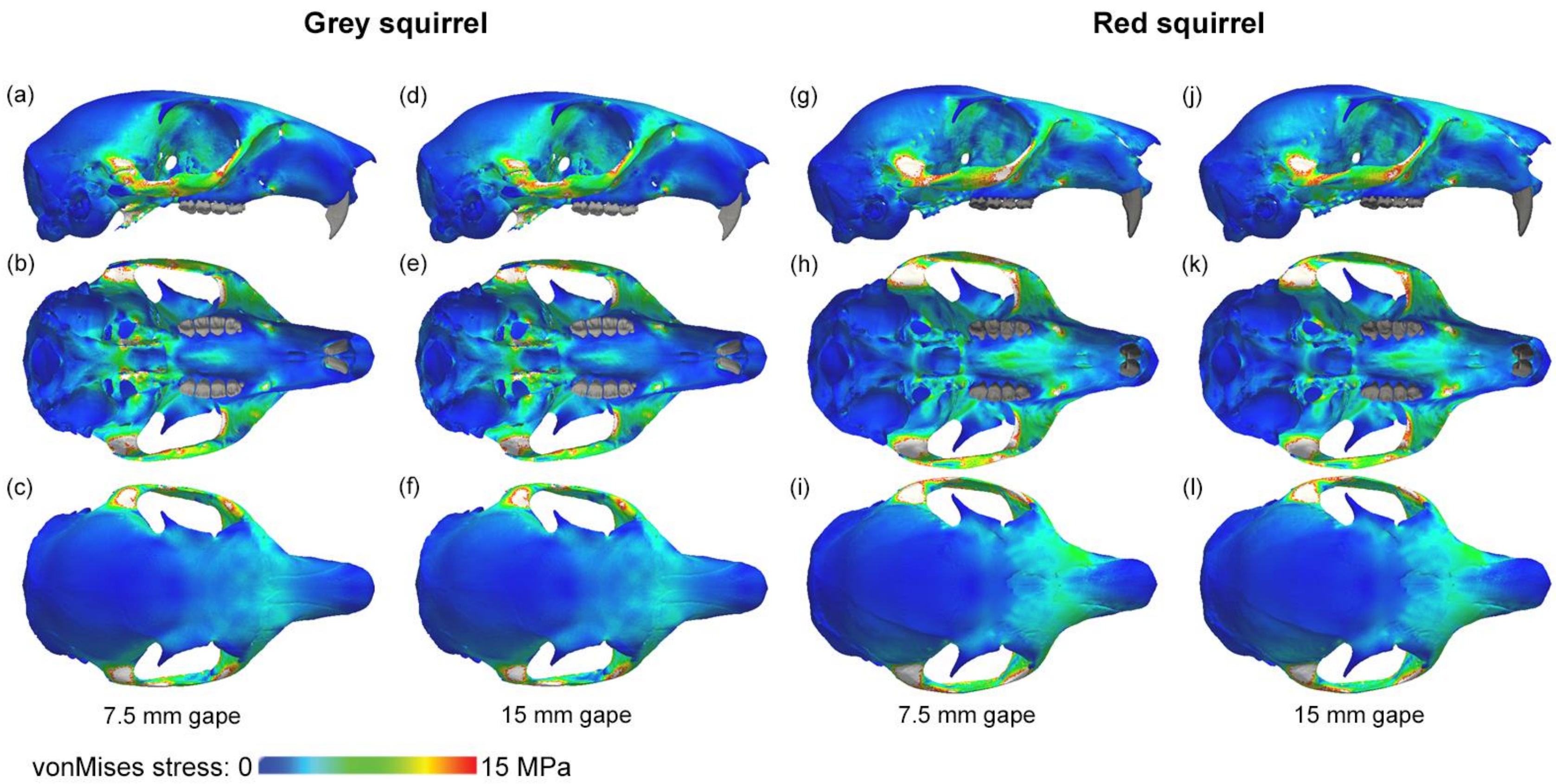

Figure S4. Contour maps showing the distribution of von Mises stresses across the skull predicted by FEA during incisor biting in (a-f) grey and (g-l) red squirrels at a gape of (a-c,g-i) 7.5 mm and (d-f,j-l) 15 mm. Views: (a,d,g,j) right lateral, (b,e,h,k) ventral, and (c,f,i,l) dorsal. Warm colours represent high stresses, cool colours represent low stresses. White areas represent elements experiencing greater than 15 MPa.
